# Reply to Chaudhuri et al. Comment on “Balwierz et al. Potential Carcinogens in Makeup Cosmetics. *Int. J. Environ. Res. Public Health* 2023, *20*, 4780”

**DOI:** 10.3390/ijerph20196902

**Published:** 2023-10-09

**Authors:** Radosław Balwierz, Paweł Biernat, Agata Jasińska-Balwierz, Dawid Siodłak, Anna Kusakiewicz-Dawid, Anna Kurek-Górecka, Paweł Olczyk, Wioletta Ochędzan-Siodłak

**Affiliations:** 1 Faculty of Chemistry, University of Opole, 45-052 Opole, Poland; dsiodlak@uni.opole.pl (D.S.); kusak@uni.opole.pl (A.K.-D.); wsiodlak@uni.opole.pl (W.O.-S.); 2 Department of Drug Forms Technology, Faculty of Pharmacy, Wroclaw Medical University, 50-556 Wroclaw, Poland; pawel.biernat@umw.edu.pl; 3 Department of Pharmacology, Academy of Silesia, 40-555 Katowice, Poland; agata.jasinska.balwierz@gmail.com; 4 Department of Community Pharmacy, Faculty of Pharmaceutical Sciences in Sosnowiec, Medical University of Silesia in Katowice, Kasztanowa 3, 41-200 Sosnowiec, Poland; akurekgorecka@sum.edu.pl (A.K.-G.); polczyk@sum.edu.pl (P.O.)

Comments by Chaudhuri et al. (2023) [1] raise several helpful points concerning the terminology utilized in the preparation of our paper and provide valuable additional data to support our publication. The viewpoint expressed by Chaudhuri et al. offers an opportunity to enhance and broaden the discussion in our article, which primarily aimed to shed light on the somewhat ambiguous and non-obvious issue of the presence of potentially carcinogenic substances in cosmetics.

Regarding the definition of carbon black, we made a simplification in our paper. We should have been more precise by specifying the source we used and cited. This source states that carbon black is a type of elemental carbon produced through controlled steam-phase pyrolysis and partial combustion of hydrocarbons [2].

We used the terms “black carbon” and “carbon” interchangeably as synonyms for carbon black, based on the information provided in reference [2]. However, we now realize that we accepted this information too hastily without fully considering the nuances. In the paper cited by us [2], particularly in Chapter “1.1.1. Nomenclature,” we found the term “The Chemical Abstract Service Registry Number for all carbon blacks is 1333–86–4.” As a result, we made some digressions and used the terms interchangeably.

As far as the abstract is concerned, there is a typo; we meant to write “IARC” instead of “IRAC”.

Similarly to the information provided above, we have referred to the IARC classification of carbon black as Group 2B, based on source [2], which is consistent with our position presented in the conclusion of Section 4.2.1. However, it is possible that we did not present this information accurately in our work. It is worth noting that incorrect classification does not eliminate the risk of carbon black’s carcinogenicity. An IARC classification of 2B is not the same as 3. In other words, the risk of cancer still exists but with much less certainty. This risk would not exist if IARC classified carbon black as Class 3. It is important to note that there is “inadequate evidence in humans” regarding the carcinogenicity of carbon black, which aligns with the viewpoint of Chaudhuri et al. [1]. It should be noted that the studies referenced as 112 [3] and 78 [4] by us, as confirmed by Chaudhuri, do not specifically address the risk associated with the lungs. These studies as lung-related risks were an over-interpretation on our part. However, these studies do provide data on the potential carcinogenicity of carbon black. Puntoni et al. [3] conclude that “Exposure to carbon black experienced by dockyard workers was associated with a two-fold increased risk of bladder cancer”. On the other hand, Wellmann et al. [4] conclude that “The mortality from lung cancer among German carbon black workers was increased,” while also noting that “results also provide little evidence for an effect of carbon black exposure.” Additionally, there are studies on air pollution that indicate the role of black carbon and polycyclic aromatic hydrocarbons (PAHs) in increasing the incidence of skin cancer [5].

Regarding the comment on carbon black and its association with dermal keratosis and leukoplakia, we should provide more specific data from Section 3.4 of reference [2], which discusses carbon black. Additionally, it is worth mentioning the evidence regarding the potential risk of carbon black as a component of tattoo dye, as mentioned in the study [6]. It is important to note that the method of application in the context of tattoo dye differs significantly from other forms of exposure.

Regarding potential eye irritation caused by carbon black, it is important to note that any cosmetic ingredient, if it comes into contact with the eyes, has the potential to cause an irritant reaction. Furthermore, when considering the use of carbon black as a component of ink, Serup states that skin reactions in black tattoos are typically papula-nodular and non-allergic, primarily related to the agglomeration of carbon black nanoparticles [7].

Chaudhuri’s mention of the SCCS position, which states that cosmetic products applied to healthy intact skin are safe, should certainly be acknowledged and adopted. It cannot be ruled out that users may apply cosmetic products to damaged skin. Damage to the protective barrier, as well as dry skin and itching, are closely related and form the foundation of many skin diseases. Damage can result from environmental, genetic, or inflammatory factors, all of which can weaken the skin’s barrier [8]. Applying cosmetic products to damaged or irritated skin can lead to allergic or irritant reactions. Moreover, applying products to compromised skin can increase the risk of bacterial or fungal infections, excessive skin dryness, hyperpigmentation, or even further damage to the skin’s barrier. Damaged skin also provides an easier pathway for the penetration of various substances. Therefore, it is important to consider other unintended uses of cosmetic products and the potential for their inhalation during the application, which cannot be definitively ruled out. Furthermore, the data presented in the article and the information provided above regarding tattoos suggest that carbon black as a cosmetic ingredient warrants attention. This was our objective when editing this chapter. Cosmetics are often used over long periods and the relationship between the skin and makeup is a complex process, especially in areas with thinner skin, such as around the eyes. This complexity can lead to potentially unintended reactions to cosmetic ingredients. We believe that Chaudhuri’s position is relevant and important in the ongoing discussion. Simultaneously, we recognize the need for further observations on carbon black and the importance of readers being aware of the published data and the ongoing discussion. Therefore, the section’s title was posed as a question, because carbon black was one of the frequently occurring ingredients in the cosmetics we analyzed. Additionally, the title of the article suggests a potential rather than unequivocal confirmation of a carcinogen affecting some cosmetic ingredients, which is due to the regulations cited by Chaudhuri et al. Thus, our standpoint should be regarded as a contribution to the discussion.
